# Revision of Korean species of the genus *Batriscenellus* Jeannel (Staphylinidae, Pselaphinae, Batrisitae) with description of one new species

**DOI:** 10.3897/zookeys.1033.59558

**Published:** 2021-04-22

**Authors:** Jun-Young Kang, Sun-Jae Park, A-Young Kim, Jong-Seok Park

**Affiliations:** 1 S1-5 302, Department of Biology, Chungbuk National University, 1 Chungdae-ro, Seowon-gu, Cheongju-si, Chungbuk-do 28644, South Korea; 2 Animal Resources Division, National Institute of Biological Resources, Incheon 22689, South Korea

**Keywords:** Batrisini, biodiversity, biogeography, rove beetles, systematics, taxonomy

## Abstract

The genus *Batriscenellus* Jeannel, 1958 (type species: *Batrisus fragilis* Sharp) includes 35 species and is known from South Korea, China, Japan, Russia, and India. Three species, *B. vicarius*, *B. auritus*, and *B. orientalis* have been documented from the Korean Peninsula. One additional species, *Batriscenellus koreanus***sp. nov.**, is described as new. Redescriptions of the Korean species, a species key, illustration of habitus, and diagnostic characters are provided.

## Introduction

The genus *Batriscenellus* Jeannel, 1958 was described based on *Batrisus fragilis* Sharp from Kioto, Japan. It includes 35 species and is known from South Korea, China, Japan, Russia, and India (Yin 2020). Three species, *B. vicarius* Löbl, 1973, *B. orientalis* (Löbl, 1973), and *B. auritus* (Löbl, 1974) are known from the Korean Peninsula. The first Korean species of the genus, *Batriscenellus japonicus vicarius* Löbl, 1973 was described from the northern part of the Korean Peninsula. It was subsequently raised to the species level by Nomura and Lee (1992). Löbl (1973, 1974) described two additional species, *Batrisiella aurita* and *Batrisiella orientalis*, also from the northern part of the Korean Peninsula, and they were transferred to *Batriscenellus* by Yin et al. (2011) and Nomura (1991), respectively.

During a revisionary study of the Korean *Batriscenellus* species, we documented four species, the previously known three species and one new species. The present paper presents the first revisionary study of Korean species of *Batriscenellus*, and describes the new species. This new species brings the number of *Batriscenellus* species from the Korean Peninsula to four.

## Materials and methods

Twenty-two specimens were examined. They are deposited in the following collections:

**CBNUIC**Chungbuk National University Insect Collection, Cheongju, Republic of Korea;

**CNUIC** Chungnam National University Insect Collection, Daejeon, Republic of Korea;

**NIBR**National Institute of Biological Resources, Incheon, Republic of Korea.

At least one specimen of each species was dissected to study male genitalia and other detailed characters. Terminology and nomenclature using descriptions follow Chandler (2001). Numbering of abdominal sclerites indicates a morphological segment. Specimen label data for the holotypes is transcribed verbatim. Data for the other specimens are standardized for consistency. Application of the terms ‘dorsal’ and ‘lateral’ to the male genitalia including the right and left apical lobe and the paramere refer to the orientation in the illustrations. The specimens were observed using a Leica M80 and MD 1000 LED optical microscope and images generated using Leica Las version 4.12 and Zerene Stacker. The map of South Korea is based on an image from SimpleMappr (Shorthouse 2010) that was subsequently modified to add locality marks.

### Key to Korean species of the genus *Batriscenellus* Jeannel

**Table:** 

1	Abdominal tergite IV or VI without depression; phallobase of male genitalia without apophysis (Fig. 8)	***Batriscenellus orientalis***
–	Abdominal tergite with depression (Figs 1D, 3C, 5C); left side of phallobase of male genitalia with apophysis posteriorly (Figs 2A, B, 4A, B, 6A, B)	**2**
2	Elytra IV or VI with a pair of processes laterally; abdominal tergite IV with sulcus (Fig. 5C); abdominal ventrite VIII without paired medial setiferous patches (Fig. 5D)	***B. auritus***
–	Elytra without processes; abdominal tergite VI with sulcus (Figs 1D, 3C); abdominal ventrite VIII with a pair of medial setiferous patches (Figs 1E, 3D)	**3**
3	Abdominal ventrites IV–VII with a pair of long setae at middle (Fig. 3D); paramere of male genitalia not bifid (Fig. 4C, D)	***B. vicarius***
–	Abdominal ventrites IV–VII without a pair of long setae at middle (Fig. 1E); paramere of male genitalia bifid (Fig. 2C, D)	***Batriscenellus koreanus* sp. nov.**

## Systematics

### Family Staphylinidae Latreille, 1802

**Subfamily Pselaphinae Latreille, 1802**

#### Supertribe Batrisitae Reitter, 1882

##### Batriscenellus

Taxon classificationAnimaliaColeopteraStaphylinidae

Jeannel, 1958 (type species: Batrisus fragilis Sharp, 1883)

6515FA27-724B-53EC-ADC4-3A1049B4270C

Batriscenellus Jeannel, 1958 (type species: Batrisus fragilis Sharp, 1883)
Batriscenellus Jeannel, 1958: 60. Löbl and Besuchet 2004: 276. Yin et al. 2011: 37.
Batriscenellinus Nomura, 1991: 321 (type species Batriscenellus uenoi Nomura, 1991).
Coreoscenellus Nomura & Lee, 1993: 12 (type species Batriscenellus brachygaster Nomura & Lee, 1993).
Nipponoscenellus Nomura, 1991: 310 (type species Batriscenellus transformis Nomura, 1991).
Scaioscenells Jeannel, 1958: 60 (type species Batrisus similis Sharp, 1883).

###### Diagnosis.

**Figure 1.**
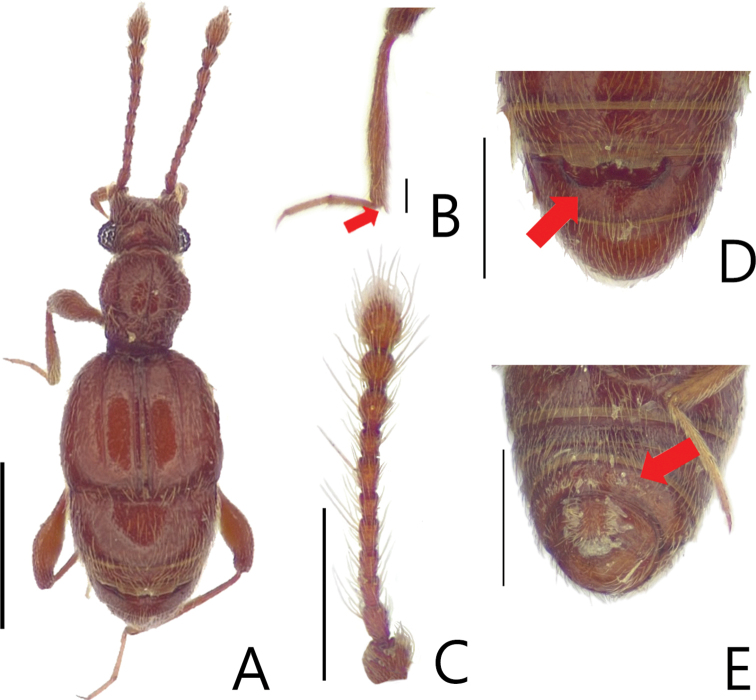
Habitus figures of *Batriscenellus koreanus* sp. nov. **A** dorsal view **B** mesotibia **C** antennae **D** dorsal view of abdomen **E** ventral view of abdomen. Scale bars: 1 mm (**A**); 0.5 mm (**B–D**).

**Figure 2.**
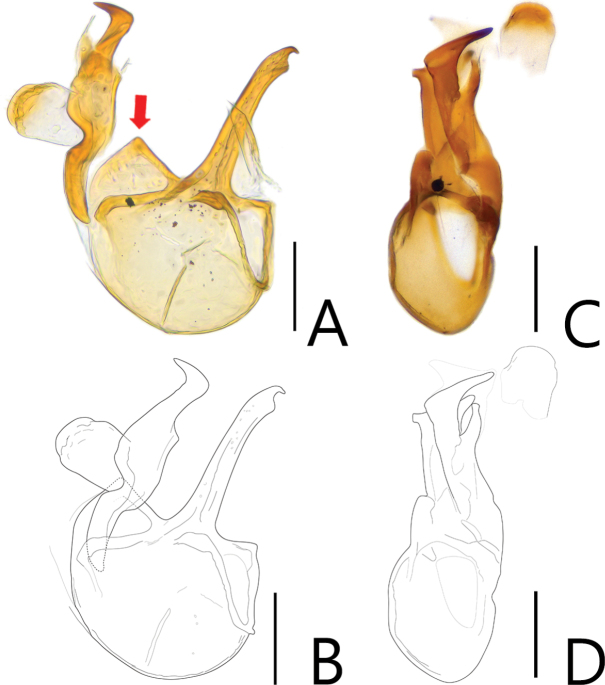
Aedeagi of *Batriscenellus koreanus* sp. nov. **A, B** dorsal view **C, D** lateral view. Scale bars: 0.1 mm.

**Figure 3.**
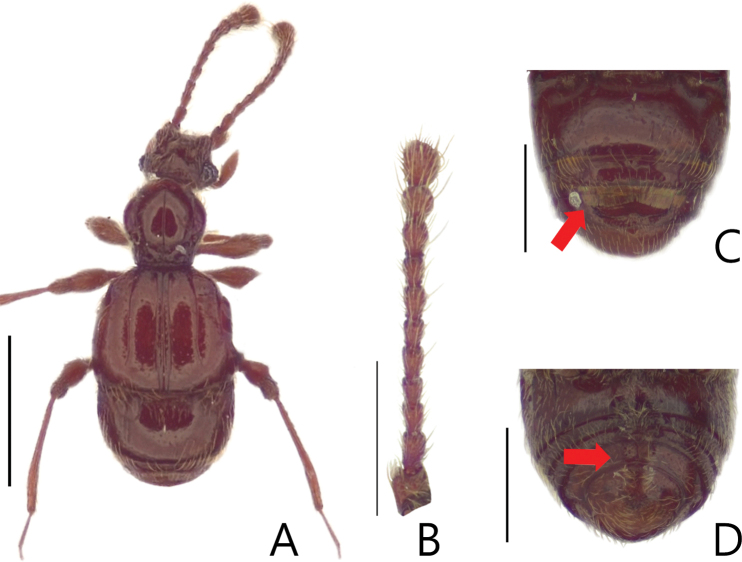
Habitus figures of *Batriscenellus vicarius***A** dorsal view **B** antennae **C** dorsal view of abdomen **D** ventral view of abdomen. Scale bars: 1 mm (**A**); 0.5 mm (**B–D**).

**Figure 4.**
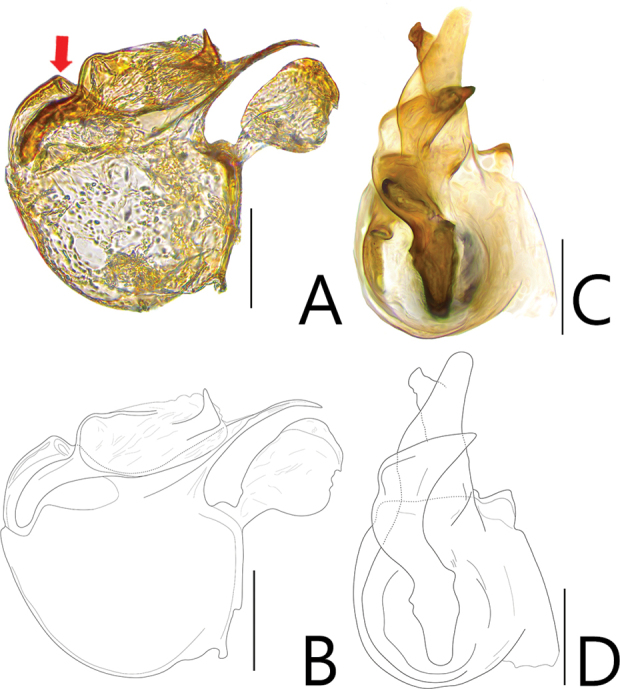
Aedeagi of *Batriscenellus vicarius***A, B** dorsal view **C, D** lateral view. Scale bars: 0.1 mm.

**Figure 5.**
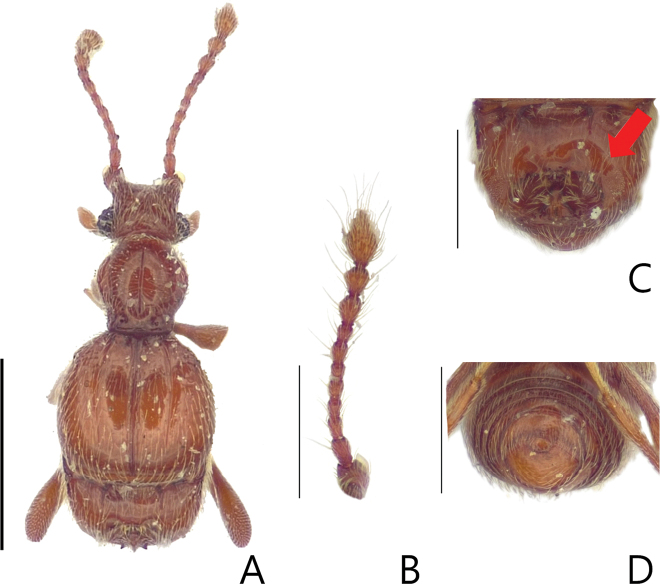
Habitus figures of *Batriscenellus auritus***A** dorsal view **B** antennae **C** dorsal view of abdomen **D** ventral view of abdomen. Scale bars: 1 mm (**A**), 0.5 mm (**B–D**).

**Figure 6.**
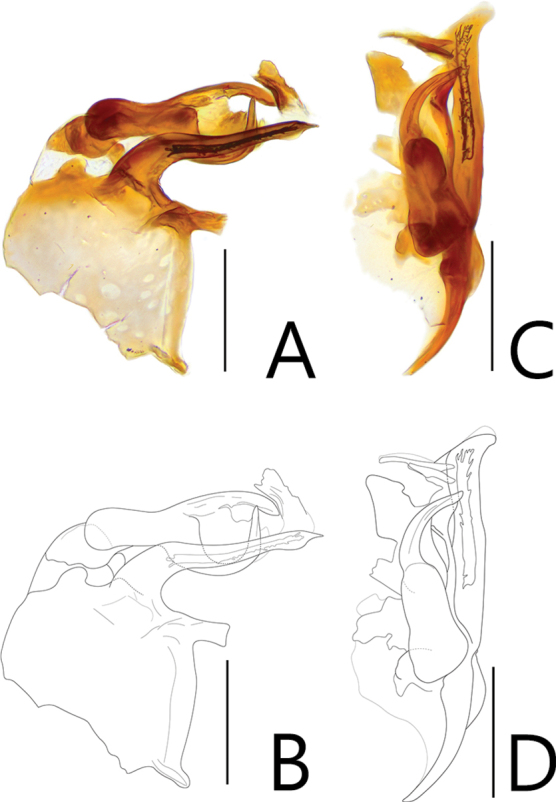
Aedeagi of *Batriscenellus auritus***A, B** dorsal view **C, D** lateral view. Scale bars: 0.1 mm.

**Figure 7.**
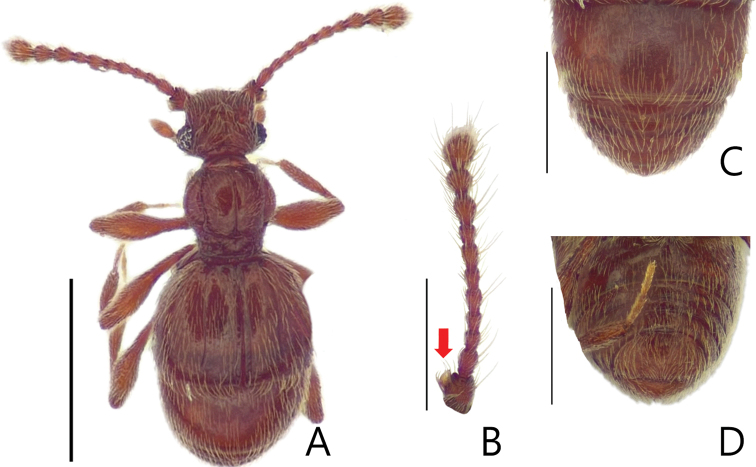
Habitus figures of *Batriscenellus orientalis***A** dorsal view **B** antennae **C** dorsal view of abdomen **D** ventral view of abdomen. Scale bars: 1 mm (**A**), 0.5 mm (**B–D**).

**Figure 8.**
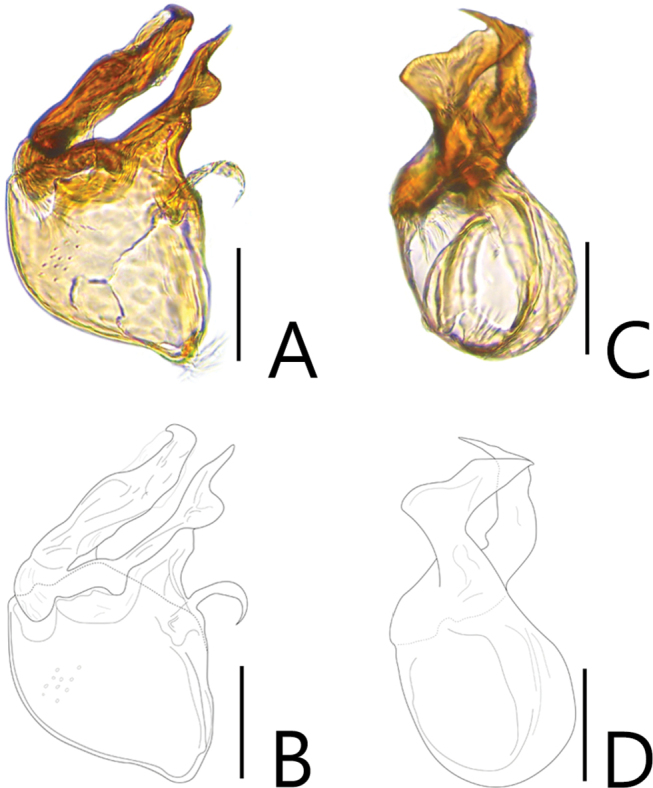
Aedeagi of *Batriscenellus orientalis***A, B** dorsal view **C, D** lateral view. Scale bars: 0.1 mm.

Members of this genus are easily separated from other genera of Batrisitae by the following combination of characters: head triangular with the transverse sulcus dorsally at mid-level of head and vertexal foveae; antennomere 1 subquadrate with dense trichomes (Figs 1C, 3B, 5B, 7B), antennomeres 9–11 clubbed; pronotum with median antebasal fovea, lateral antebasal foveae, inner basolateral foveae and outer basolateral foveae, disc with median and lateral longitudinal sulci; elytra with two basal foveae; mesotibia with spine on distal margin (Fig. 1B); abdomen rounded laterally; abdominal tergite IV largest; male genitalia asymmetric (Figs 2, 4, 6, 8); paramere singular and originating from ventral phallobase.

###### Distribution.

South Korea, Russia (Far East), Japan, China.

##### Batriscenellus koreanus

Taxon classificationAnimaliaColeopteraStaphylinidae

Kang, Park, Kim & Park sp. nov.

D00637B2-FBA0-59D6-90C4-4F789776AB90

http://zoobank.org/6E280D51-FC3F-4AE7-9A2C-9AAF2132BC74

Figs 1 , 2

###### Material examined.

***Holotype*.** 1♂ (NIBR), “Korea: Chungbuk prov. / Cheongwon-gun, / Bugi-myeon, Hwasang-ri, / 12III2020, 36°44'08.00"N, 127°29'01.40"E, 38 m / sifting soil litter / M-S Jang, / T-Y Jang”. ***Paratype*** (1 male). 1♂ (CBNUIC), same data as holotype.

###### Diagnosis.

This species can be distinguished from the other *Batriscenellus* species by the following combination of characters: antennomere 8 subquadrate and smallest (Fig. 1C), abdominal tergite V with a pair of median setiferous patches, VI with deep mediobasal sulcus (Fig. 1D), abdominal ventrite VIII with depressionand pair of dense setiferous patches (Fig. 1E), phallobase of male genitalia widely expanded, apical lobe of male genitalia curved to right in lateral view (Fig. 2C, D), paramere forked into two branches in lateral view, major branch curved to right (Fig. 2C, D).

###### Description.

Length 2.03–2.15 mm. Body reddish-brown (Fig. 1A). ***Head*.** All antennomeres with tubercles and long setae (Fig. 1C). Antennomere 1 subquadrate with dense trichomes on lateral margin, 2–7 rectangular, 8 rectangular [from photo] and smallest, 9 rectangular and larger than 3–8, 10 rhombic, 11 oval. ***Thorax*.** Each elytron with shallow discal stria. ***Abdomen*.** Abdominal tergite V with pair of setiferous patches, VI with deep sulcus (Fig. 1D: arrow). Abdominal ventrite VIII with depression and pair of dense setiferous patches (Fig. 1E: arrow). ***Aedeagus*.** Left side of phallobase widely expanded in dorsal view (Fig. 2A, B: arrow). Apical lobe of male genitalia curved right in lateral view (Fig. 2C, D). Paramere forked into two branches in lateral view, major branch curved to right (Fig. 2C, D).

###### Distribution.

**Figure 9.**
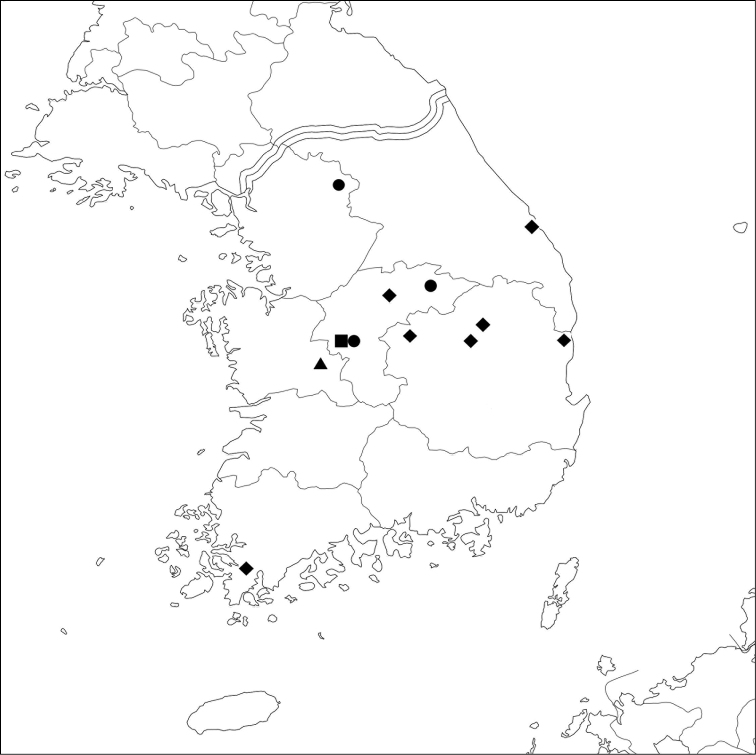
Collection localities of *Batriscenellus koreanus* sp. nov.: square; *B. vicarius*: circle; *B. auritus*: triangle; *B. orientalis*: diamond.

South Korea (Fig. 9: square)

###### Etymology.

This species is named for Korea, where this species was collected.

###### Habitat.

The two specimens of this species were collected by sifting soil litter of a riverside grassland.

##### Batriscenellus vicarius

Taxon classificationAnimaliaColeopteraStaphylinidae

Löbl, 1973

BD70C4C1-2465-51CA-8DEF-2D724E8EFAD4

Figs 3 , 4

Batriscenellus japonicus vicarius Löbl, 1973: 322. Nomura 1991: 301.
Batriscenellus vicarius : Nomura 1992: 61. Cho and Ahn 2001: 53. Park et al. 2013: 123. Löbl and Besuchet 2004: 276. Schülke and Smetana 2015: 367.

###### Material examined.

1♂ (CBNUIC), Chungbuk Prov., Cheongwon-gun, Bugi-myeon, Hwasang-ri, 38 m, 36°44'08.00"N, 127°29'01.40"E, 12 III 2020, M-S Jang, T-Y Jang, sifting soil litter; 1♂1♀ (1♂ aedeagus dissected and mounted in Euparal on clear plastic card, CBNUIC), Gyeonggi Prov., Baekdun-ri, Mt. 15–35, Buk-myeon, Gapyeong-gun, 509 m, 37°55'10.50"N, 127°26'21.80"E, 13 X 2019, J-Y Kang, J-W Kim, sifting leaf & soil litter; 1♀ (CBNUIC), Gyeonggi Prov., Baekdunro-gil 650, Buk-myeon, Gapyeong-gun, 503 m, 37°55'09.80"N, 127°26'22.50"E, 13 X 2019, M-H Song, U-J Byeon, sifting leaf & soil litter; 1♀ (CBNUIC), Gyeonggi Prov., Baekdun-ri, Buk-myeon, Gapyeong-gun, 440 m, 37°54'57.40"N, 127°26'17.20"E, 13 X 2019, J-W Kang, M-H Song, U-J Byeon, T-Y Jang, sifting leaf & soil litter; 1♂ (CBNUIC), Chungbuk Prov., Danyang-gun, Danyang-eup, Yangbangsan-gil, 585 m, 36°58'14.20"N, 128°22'57.60"E, 12 III 2020, M-S Jang, J-W Kim, sifting leaf & soil litter.

###### Diagnosis.

This species can be distinguished from the other *Batriscenellus* species by the following combination of characters: antennomeres 2–8 rectangular (Fig. 3B); abdominal tergite V with pair of basolateral setiferous patches, VI with deep sulcus (Fig. 3C: arrow); abdominal ventrites IV–VII with pair of long setae at middle (Fig. 3D), VIII with carina and pair of dense medial setiferous patches (Fig. 3D: arrow); left side of phallobase of male genitalia widely expanded in dorsal view (Fig. 4A, B: arrow); paramere of male genitalia curved to left in dorsal view (Fig. 4A, B).

###### Description.

Length 1.85–2.02 mm. Body reddish-brown (Fig. 3A). ***Head*.** All antennomeres with tubercles and long setae (Fig. 3B). Antennomere 1 subquadrate with dense trichomes laterally 2–8 rectangular, 9 rectangular and larger than 2–8, 10 rhombic, 11 oval. ***Thorax*.** Pronotum with medial and lateral longitudinal sulci. Mesoventrite with lateral setiferous patches. Each elytron with shallow discal stria. ***Abdomen*.** Abdominal ventrites IV–VII with pair of long setae distinct located at middle, distinct in female (Fig. 3D: arrow), IV with setiferous patches at posterior margin of coxal cavity of hind leg (Fig. 3D). ***Aedeagus*.** Left side of phallobase of male genitalia widely expanded in dorsal view (Fig. 4A, B: arrow). Paramere of male genitalia curved to left in dorsal view (Fig. 4A, B).

###### Distribution.

South Korea (Fig. 9: circle), Russia (Far East), Japan, China.

###### Habitat.

Specimens of this species were collected by sifting soil or leaf litter.

##### Batriscenellus auritus

Taxon classificationAnimaliaColeopteraStaphylinidae

(Löbl, 1974)

8E93943B-E38A-5268-AF1D-C32C2B7BBC55

Figs 5 , 6

Batrisiella aurita Löbl, 1974: 92. Nomura and Lee 1993: 46. Kim et al. 1994: 144. Cho and Ahn 2001: 53. Löbl and Besuchet 2004: 277.
Batriscenellus auritus : Yin et al. 2011: 37. Park et al. 2013: 123. Schülke and Smetana 2015: 366.

###### Material examined.

1♂ (1♂ aedeagus dissected and mounted in Euparal on clear plastic card, CNUIC), Chungnam Prov., Gongju City, Mt. Gyeryongsan, Geumsubong, 23 VI 2000, H.-J. Kim, ex near stream.

###### Diagnosis.

This species can be distinguished from the other *Batriscenellus* species by the following combination of characters: antennomere 1 subquadrate with dense yellowish trichomes on lateral margin; elytra with pair of process antero-laterally; abdominal tergite IV with deep mediobasal sulcus (Fig. 5C); abdominal ventrite IV with dorsolateral setiferous patches; paramere of male genitalia forked into two branches, right paramere curved to right in dorsal view (Fig. 6A, B).

###### Description.

Length 1.85 mm. Body reddish-brown (Fig. 5A). ***Head*.** All antennomeres with tubercles and long setae (Fig. 5B). Antennomere 1 subquadrate with dense yellowish trichomes on lateral margin, 2–7 rectangular, 8 subquadrate and smallest, 9–10 rhombic, 11 oval. ***Thorax*.** Mesoventrite with lateral setiferous patches. Elytra with lateral process. ***Abdomen*.** Abdominal tergite IV with deep sulcus (Fig. 5C: arrow). Abdominal ventrite IV with pair of dorsolateral setiferous patches. ***Aedeagus*.** Apical lobe of male genitalia curved to right and expanded apical margin in dorsal view (Fig. 6A, B). Two branches of paramere curved to right in lateral view (Fig. 6C, D).

###### Comments.

The basal bulb of the male genitalia are broken in Figure 6D. See Löbl (1974: 93) for other examples of the aedeagus.

###### Distribution.

South Korea (Fig. 9: triangle).

###### Habitat.

A single specimen of this species was collected near a stream.

##### Batriscenellus orientalis

Taxon classificationAnimaliaColeopteraStaphylinidae

(Löbl, 1973)

4D068DE4-2793-5E81-A45E-98A094E5C324

Figs 7 , 8

Batrisiella orientalis Löbl, 1973: 322.
Batriscenellus orientalis : Nomura 1991: 317. Kim et al. 1994: 144. Cho and Ahn 2001: 53. Löbl and Besuchet 2004: 276. Shao et al. 2010: 67. Yin et al. 2011: 37. Park et al. 2013: 123. Schülke and Smetana 2015: 366.
Batriscenellus (Coresoscenellus) brachygaster Nomura & Lee, 1993: 13. Nomura 2005: 214.

###### Material examined.

3♂♂ (1♂, aedeagus dissected and mounted in Euparal on clear plastic card, CBNUIC), Gangwon Prov., Jungyeong-gil, Miro-myeon, Samcheok-si, 69 m, 37°22'02.80"N, 129°05'06.60"E, 22 VIII 2018, Y-J Choi, light trap; 1♂ (CBNUIC), Chungbuk Prov., Jecheon-si, Hansu-myeon, Songgye-ri, 258 m, 36°52'53.40"N, 128°05'06.80"E, 23 V 2019, Y-J Choi, sifting litter near stream; 1♀ (CBNUIC), Chungbuk Prov., Mt. Worak, Mireuksonggye-ro, Hansu-myeon, Jecheon-si, 220 m, 36°52'07.60"N, 128°05'10.80"E, 14 VI 2018, Y-J Choi, sifting litter; 1♂ (CBNUIC), Gyeongbuk Prov., Uljin-gun, Onjeong-myeon, Woeseonmi-ri, 592 m, 36°45'28.30"N, 129°18'05.30"E, 9 VIII 2018, J-W Kang, sifting leaf litter; 1♂ (CBNUIC), Gyeongbuk Prov., Mungyeong-si, Sanyang-myeon, Sinjeon-ri, 74 m, 36°36'16.00"N, 128°15'47.00"E, 9 V 2019, U-J Byeon, M-H Song, sifting leaf litter; 2♀♀ (CBNUIC), Jeonnam Prov., Haenam-gun, Gyegok-myeon, Dangsan-ri, 211 m, 34°40'53.00"N, 126°38'56.00"E, 18 V 2019, J-S Park, M-H Song, leaf litter & dead wood debris; 1♀ (CBNUIC), Gyeongbuk Prov., Yeongju-si, Munsu-myeon, Wolho-ri, 172 m, 36°45'45.61"N, 128°37'25.73"E, 4 V 2019, M-S Jang, sifting leaf litter; 1♀ (CBNUIC), Gyeongbuk Prov., Yecheon-gun, Yongmun-myeon, Sanggeumgok-ri, 220 m, 36°41'51.00"N, 128°24'18.00"E, 5 V 2019, U-J Byeon, sifting leaf litter; 1♀ (CBNUIC), Gyeongbuk Prov., Yecheon-gun, Yongmun-myeon, Nosa-ri, 246 m, 36°40'57.00"N, 128°22'31.00"E, 19 VII 2019, U-J Byeon, sifting leaf & soil litter.

###### Diagnosis.

This species can be distinguished from the other *Batriscenellus* species by the following combination of characters: antennomere 1 subquadrate with dense yellowish trichomes; elytra with lateral process; right margin of apical lobe of male genitalia expanded in dorsal view (Fig. 8A, B); paramere of male genitalia curved to left in dorsal and lateral views (Fig. 8C, D).

###### Description.

Length 1.71–2.09 mm. Body reddish-brown (Fig. 7A). ***Head*.** All antennomeres with tubercles and long setae (Fig. 7B). Antennomere 1 subquadrate with dense yellowish trichomes on lateral margin (Fig. 7B: arrow), 2–7 rectangular, 8 subquadrate and smallest, 9 rhombic, 10 subquadrate, 11 oval. ***Thorax*.** Mesoventrite with pair of lateral setiferous patches. Each elytron with one discal stria. ***Abdomen*.** Abdominal tergite IV expanded (Fig. 5C). Abdominal ventrite IV with lateral setiferous patches.

###### Distribution.

South Korea (Fig. 9: diamond), Japan, China.

###### Habitat.

Most specimens of this species were collected by sifting leaf litter or dead wood debris. One specimen was captured by a light trap.

## Supplementary Material

XML Treatment for Batriscenellus

XML Treatment for Batriscenellus koreanus

XML Treatment for Batriscenellus vicarius

XML Treatment for Batriscenellus auritus

XML Treatment for Batriscenellus orientalis
